# Western Juniper Management: Assessing Strategies for Improving Greater Sage-grouse Habitat and Rangeland Productivity

**DOI:** 10.1007/s00267-015-0521-1

**Published:** 2015-05-10

**Authors:** Shahla Farzan, Derek J. N. Young, Allison G. Dedrick, Matthew Hamilton, Erik C. Porse, Peter S. Coates, Gabriel Sampson

**Affiliations:** 1 Department of Entomology, University of California, Briggs Hall, One Shields Avenue, Davis, CA 95616 USA; 2 Department of Plant Sciences, University of California, Davis, USA; 3 Department of Environmental Science and Policy, University of California, Davis, USA; 4 Department of Civil and Environmental Engineering, University of California, Davis, USA; 5 U.S. Geological Survey, Western Ecological Research Center, Dixon Field Station, 800 Business Park Drive, Suite D, Dixon, CA 95620 USA; 6 Department of Agricultural and Resource Economics, University of California, Davis, USA

**Keywords:** *Centrocercus urophasianus*, *Juniperus occidentalis* subsp. *occidentalis*, Multi-objective management, Optimization modeling, Resource management, U.S. Endangered Species Act

## Abstract

Western juniper (*Juniperus occidentalis* subsp. *occidentalis*) range expansion into sagebrush steppe ecosystems has affected both native wildlife and economic livelihoods across western North America. The potential listing of the greater sage-grouse (*Centrocercus urophasianus*) under the U.S. Endangered Species Act has spurred a decade of juniper removal efforts, yet limited research has evaluated program effectiveness. We used a multi-objective spatially explicit model to identify optimal juniper removal sites in Northeastern California across weighted goals for ecological (sage-grouse habitat) and economic (cattle forage production) benefits. We also extended the analysis through alternative case scenarios that tested the effects of coordination among federal agencies, budgetary constraints, and the use of fire as a juniper treatment method. We found that sage-grouse conservation and forage production goals are somewhat complementary, but the extent of complementary benefits strongly depends on spatial factors and management approaches. Certain management actions substantially increase achievable benefits, including agency coordination and the use of prescribed burns to remove juniper. Critically, our results indicate that juniper management strategies designed to increase cattle forage do not necessarily achieve measurable sage-grouse benefits, underscoring the need for program evaluation and monitoring.

**Electronic supplementary material:**

The online version of this article (doi:10.1007/s00267-015-0521-1) contains supplementary material, which is available to authorized users.

## Introduction

Over the last 130 years, western juniper (*Juniperus occidentalis* subsp. *occidentalis*) populations have expanded into large areas of sagebrush steppe habitat across western North America (Miller et al. 2000; Davies et al. 2011). A mix of environmental and managerial factors in the landscape have facilitated this range expansion, including fire suppression (Miller et al. 2000), fuel reductions from grazing (Burkhardt and Tisdale 1976; Miller and Rose 1995), and increased atmospheric carbon dioxide (Soulé et al. 2004).

**Fig. 1:**
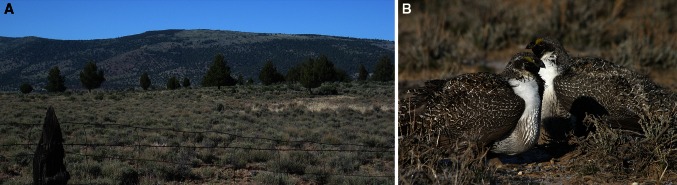
**a** Western juniper (*Juniperus occidentalis* subsp. *occidentalis*) in Modoc County, CA. Photo credit: Allison G. Dedrick. **b** A pair of male greater sage-grouse (*Centrocercus urophasianus*). Photo credit: Gail Patricelli

In sagebrush ecosystems, juniper range expansion (Fig. 1a) threatens both native wildlife and agricultural productivity (Miller et al. 2000; Bates 2005; Noson et al. 2006). For example, the conversion of sagebrush steppe to juniper woodland negatively affects greater sage-grouse (*Centrocercus urophasianus*, Fig. 1b) by reducing sagebrush cover and the associated plants and insects that comprise the birds’ diet (Crawford et al. 2004; Doherty et al. 2008; Baruch-Mordo et al. 2013). In addition, changes in the geographic range and density of juniper can affect forage for cattle. In the Great Plains region, livestock production has dropped by 75 % in areas where the closely related eastern red-cedar (*Juniperus virginiana*) has encroached into grasslands (Twidwell et al. 2013).

To address the impact of western juniper expansion on greater sage-grouse and sustainable grazing, the Natural Resources Conservation Service (NRCS) launched the Sage-Grouse Initiative (SGI) in 2010 (NRCS 2012a). SGI funds juniper removal and sagebrush steppe habitat restoration projects on public and private lands with the goal of simultaneously improving environmental and economic objectives (NRCS 2012b). Despite a decade of initiatives and funding, as well as the pending listing decision for the greater sage-grouse (hereafter sage-grouse) under the U.S. Endangered Species Act (USFWS 2010), few studies have assessed the implementation of juniper removal strategies (Baruch-Mordo et al. 2013). Sage-grouse habitat restoration is often promoted as complementary with cattle grazing (NRCS 2012a), but the degree of complementarity has not been evaluated in the scientific literature.

Multi-objective management of natural resources seeks to balance human demands, environmental preservation, and future resource availability (Schmoldt 2001). Decision-makers must often prioritize among different goals by evaluating the economic and environmental benefits of various actions. Software tools such as *Zonation* (Williams et al. 2005) and *Marxan* (Possingham et al. 2000) can support decision-making for landscape-level conservation planning, but limited resources and learning curves often restrict extensive use of such tools by land managers. Additionally, these off-the-shelf programs may not be applicable to certain management tasks due to issues of model formulation (cost minimization or data structure) or focus (marine reserves, land parcels, etc.). Simpler, more adaptable tools can allow decision-makers to more rapidly assess strategies for conservation and resource management.

We developed a spatially explicit decision model using multi-objective optimization to assess juniper management strategies for (1) sage-grouse habitat restoration and (2) forage production for cattle. Our primary goal was to assess whether juniper management programs designed to improve sage-grouse habitat can yield forage production benefits and vice versa. Although land availability and owner willingness currently drive site selection for SGI projects, we explored how land managers could prioritize locations based on their suitability for one or both objectives. Our research provides a timely evaluation of SGI management strategies and contributes to the growing collection of integrated modeling tools for conservation of threatened species and resource management.

## Methods

**Fig. 2:**
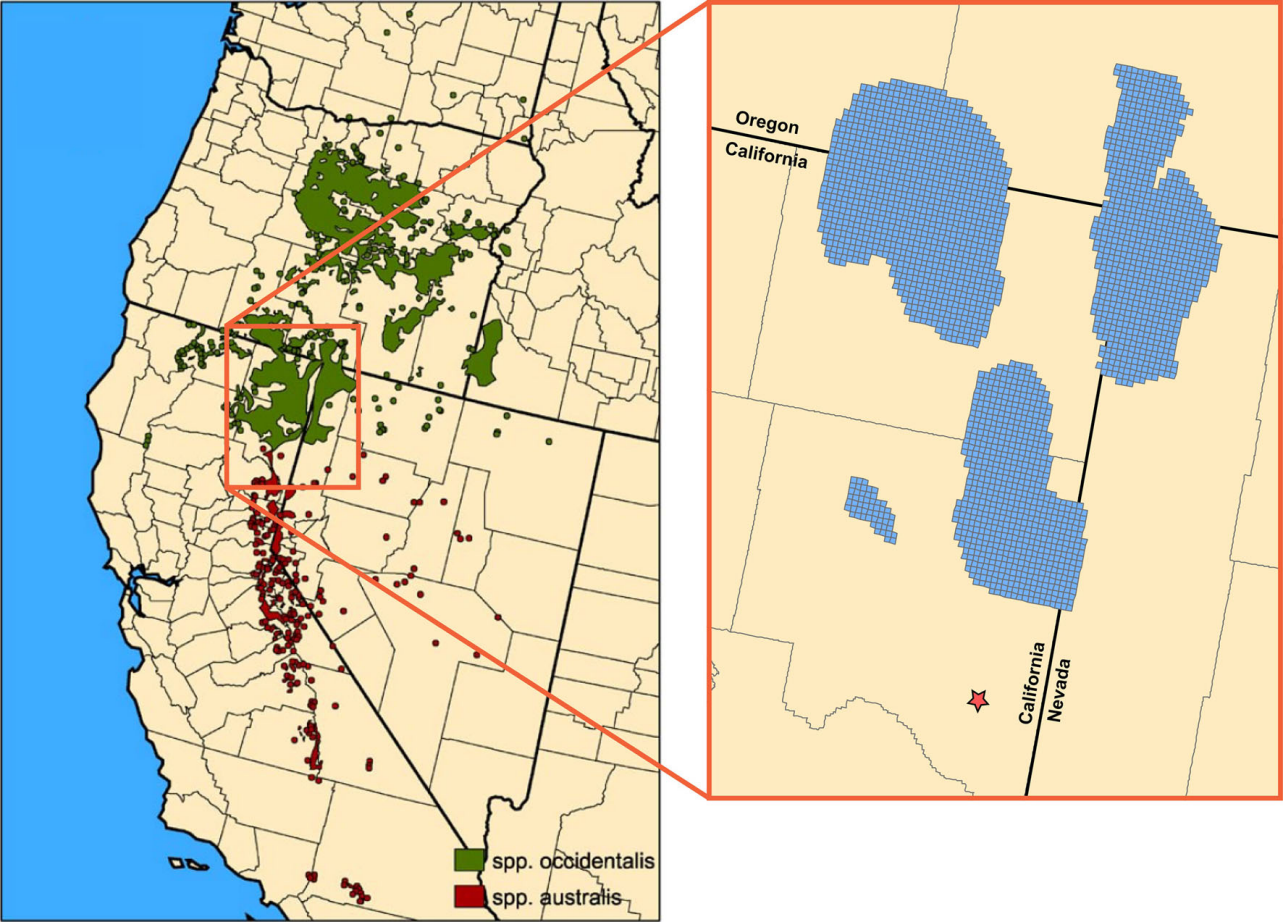
Distribution of western juniper (*Juniperus occidentalis* subsp. *occidentalis*, shown in *green*) modified from Miller et al. 2005. *Inset map* indicates study region, with *blue* 2000 × 2000 m grid cells representing “decision units” considered in the model and the location of the Greenleaf Power Plant in Wendel, CA (*red star*)

We analyzed optimal budget allocations for juniper removal across a region of the Modoc Plateau (Fig. 2) using a simple and novel algorithm that adapted several optimization approaches. We used a ranking procedure to sequentially select the best available sites for treatment (DeVore and Temlyakov 1996) and developed a procedure for incorporating multiple goals with different units derived from the “constraint method” in linear programming (Haimes 1970). The analysis optimized juniper removal decisions across a landscape, assessing costs and benefits to identify the best areas for treatment. We then extended the analysis to include several alternative cases. The section below describes: (1) methods for determining benefits and costs, (2) model formulation and implementation, and (3) alternative analysis cases.

### Benefits of Treatment: Forage Production

We obtained herbaceous vegetation and juniper canopy cover data from Coultrap et al. (2008) for 97 circular plots (45 m diameter) in Modoc, Lassen, and Siskiyou counties. Based on a comparison of sites with intact western juniper communities and those where juniper was removed, Coultrap et al. (2008) reported that juniper removal led to increased grass cover, higher herbaceous productivity, and less bare ground. The authors chose study plots that: (1) were representative of soil and vegetation types in the area, (2) exhibited variable juniper canopy cover, (3) had not been grazed, and (4) allowed for comparison of treated sites (juniper removed) and adjacent untreated sites. Mean juniper canopy cover across the 97 sites was 12 % (range 0–74 %).

For each plot location, we assessed a range of environmental and geophysical variables as potential predictors of forage production, including date of onset of the frost-free period, temperature difference between the average warmest and coldest month, soil properties, slope, and elevation (Appendix A1). We obtained juniper canopy cover data from an NRCS analysis of aerial photography (Falkowski and Evans 2012). We derived downscaled climate data from the PRISM dataset (PRISM Climate Group 2012) using the ClimateWNA tool (Wang et al. 2011). We obtained soil and topographic information from the NRCS STATSGO2 database (NRCS 2012c) and ASTER DEM data (EOSDIS 2009), respectively. Adopting the common log-linear regression specification (Johnson et al. 1999), we used these environmental and geophysical variables to estimate the relationship between forage production and juniper canopy cover in the original plots from the Coultrap et al. (2008) dataset. We then applied this function to estimate potential increases in forage production following complete juniper removal for sites across the entire study region (Appendix A1). While we use the term “forage” to refer to all herbaceous plant material, we recognize that not all herbaceous plants provide equal nutritional benefit for cattle.

### Benefits of Treatment: Sage-grouse

We assessed benefits of juniper treatment to sage-grouse populations using a spatially explicit model to predict the relative probability of sage-grouse occurrence based on density of breeding birds observed near leks. Leks are ideal locations for space use analyses because they are hubs for nesting (Autenrieth 1985; Connelly et al. 2004) and are generally centered among seasonal use areas (Coates et al. 2013).

To calculate a “breeding density index”, we obtained lek coordinates and count data (number of males attending leks) from the California Department of Fish and Wildlife and the Oregon Department of Fish and Wildlife. We used a kernel density estimator (Silverman 1986) with smoothing parameters estimated using likelihood based cross-validation to create a utilization distribution (“UD”, Appendix A2). Given the distribution and density of documented animal occurrences, UDs provide an approximation of sage-grouse space use (Coates et al. 2013). We then used the UD to calculate a “dispersal index” representing the probability of sage-grouse occurrence in each landscape cell given complete juniper removal within that cell. For additional detail on methodologies used to assess sage-grouse benefits, see Appendix A2.

### Costs of Treatment

To collect data on treatment costs as well as decision-making heuristics for treatment method selection, we interviewed stakeholders with firsthand experience implementing juniper treatment projects on the Modoc Plateau (UC Davis IRB #420715-1). Respondents included representatives from private consulting firms, federal agencies, and cooperative extension. The aggregated data from these interviews provided us with a range of thresholds to inform treatment method selection, including the maximum juniper canopy cover and slope for various treatment methods, as well as the per hectare cost of using various methods (Tables A2 and A3).

We considered the two most commonly used methods of juniper removal: *hand treatment,* which involves felling trees with chainsaws in regions of low juniper density, and *mechanical treatment,* which uses heavy machinery to fell and pile trees and is most effective at high juniper density (Table A3). We did not allow treatment in: (1) areas with >30 % slope (averaged at the one hectare scale) due to reported concerns about accessibility and post-treatment erosion or (2) areas with >30 % juniper cover due to sparse understory cover and a depauperate seed bank (Miller et al. 2005). Based on interview responses, we assigned hand treatment a fixed cost of $100/ha and mechanical treatment a cost of $300/ha (Table A2).

To compare different treatment regimes, we conducted analyses assuming a budget of $5 million for the study region. This value roughly corresponds to SGI funding allocated to projects on the Modoc Plateau ($5.9 million) during the 2011 fiscal year (NRCS 2012b). Because our study region does not include the entire Modoc Plateau, we rounded down the SGI budget to obtain a more realistic funding value for the area.

### Model Implementation

The model is a simplified Greedy Algorithm (DeVore and Temlyakov 1996) implemented in the R Statistical Environment (R Development Core Team 2014). Each cell in the grid has attributes for treatment cost, treatment benefit for sage-grouse habitat, and treatment benefit for forage production. The model assumes that when any given cell is treated, all trees are removed within the cell. The algorithm calculates the *weighted cost*-*effectiveness* ($Z_{i}$) for a cell $i$ as the total weighted benefits of treating a cell divided by the cost ($C_{i}$) of treating that cell:

1 $$Z_{i} = \frac{{\left[ {\left( {W_{\text{forage}} * B_{{i_{\text{forage}} }} } \right) + \left( {W_{\text{habitat}} * B_{{i_{\text{habitat}} }} } \right)*f} \right]}}{{C_{i} }}$$

Each cell has an associated benefit from treatment for forage production ($B_{{i_{\text{forage}} }}$) and sage-grouse habitat ($B_{{i_{\text{habitat}} }}$). The forage-to-habitat conversion factor ($f$) creates comparable values between the two goals:

2 $$f = \frac{{B_{{{ \hbox{max} }_{\text{forage}} }} }}{{B_{{{ \hbox{max} }_{\text{habitat}} }} }}$$

In Eq. 2, the maximum forage benefit ($B_{{{ \hbox{max} }_{\text{forage}} }}$), which is measured in kilograms, is the benefit achievable within a given budget when only maximizing forage production. Similarly, the maximum habitat benefit ($B_{{{ \hbox{max} }_{\text{habitat}} }}$) is the benefit achievable within the same budget when only maximizing habitat. Sage-grouse habitat benefits are measured as a percentage of the total benefits possible if all sage-grouse habitat improvements in the study region were made. Finally, the two weights are applied as percentages:

3 $$W_{\text{habitat}} = 1 - W_{\text{forage}}$$

**Fig. 3:**
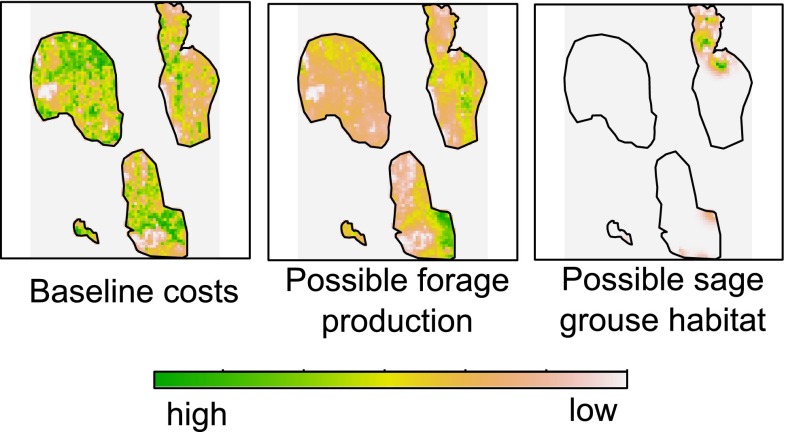
Heat map of treatment costs, forage production benefits, and sage-grouse habitat benefits in the study area (Modoc County, CA). *Green* indicates high values, while *pink* and *white* designate low values

For each case, we ran the algorithm multiple times, each with different values of $W_{\text{forage}}$ and $W_{\text{habitat}}$ ranging from 0 to 1 and 1 to 0, respectively. The algorithm calculates the weighted cost-effectiveness of each cell based on the given weighting factors, ranks the cells from high to low based on weighted cost-effectiveness, and iteratively selects to treat the most cost-effective untreated cell until the budget limit is reached. The result is a spatially explicit map of treated cells and total estimated benefits for both sage-grouse and livestock that corresponds to a given budget (Fig. 3). Our study region consisted of 877,200 ha, divided into 2193 treatment sites of 4 km^2^ each.

### Alternative Cases

We carried out several alternative cases that either introduced other factors or relaxed assumptions in our baseline model about a key ecological, economic, or administrative condition (Table A3). Our alternative cases, detailed in Appendix section A.4, were: (1) degree of coordination among land management groups, (2) sale of chipped juniper biomass as an offset to treatment cost, (3) variable budget constraints, and (4) fire as a juniper treatment method.

## Results

**Fig. 4:**
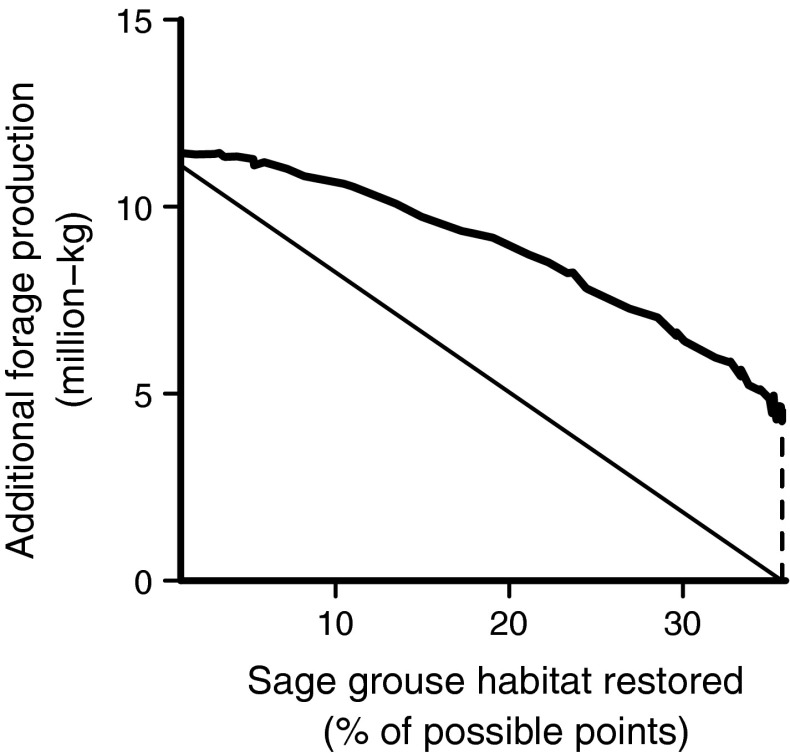
Tradeoffs between prioritizing forage production and sage-grouse habitat restoration, as illustrated by the efficiency frontier for the baseline scenario. The *straight line* indicates the frontier that would exist if forage production and sage-grouse habitat had a perfect tradeoff (i.e., if choosing one goal achieved none of the other). The baseline efficiency frontier lies above the perfect tradeoff line, suggesting some synergy between forage production and sage-grouse habitat goals. The *dashed line* connecting the baseline efficiency frontier to the x-axis shows the amount of forage production gained when sage-grouse goals are prioritized 100 %. Completely prioritizing forage production achieves about 1 % of sage-grouse habitat restoration, but is not visible on the graph

Our results suggest that sage-grouse habitat and forage production benefits: (1) are sometimes complementary, (2) exhibit decreasing-returns-to-scale, and (3) depend on landscape characteristics. There are several potential relationships between the two goals, illustrated in Fig. 4. These include a hypothetical 1:1 tradeoff in sage-grouse habitat and forage production (straight line) and the model results (labeled curve) (Fig. 4). We refer to these curves as *efficiency frontiers* (Polasky et al. 2008). Each curve shows the maximum production for a given budget, while the concavity of the model results curve indicates tradeoffs in the two goals with decreasing-returns-to-scale (Fig. 4). In this case, decreasing-returns-to-scale refers to the scenario in which juniper treatment increases by a factor *m,* but outputs (either sage-grouse or forage production benefits) increase by less than *m*. Alternatively, moving away from the intersection between the labeled curve and the y-axis, an initial *m* reduction in forage production results in greater than *m* gains in sage-grouse habitat restoration (Fig. 4). For each successive *m* reduction in forage production, the corresponding increase in sage-grouse benefits gets smaller.

**Fig. 5:**
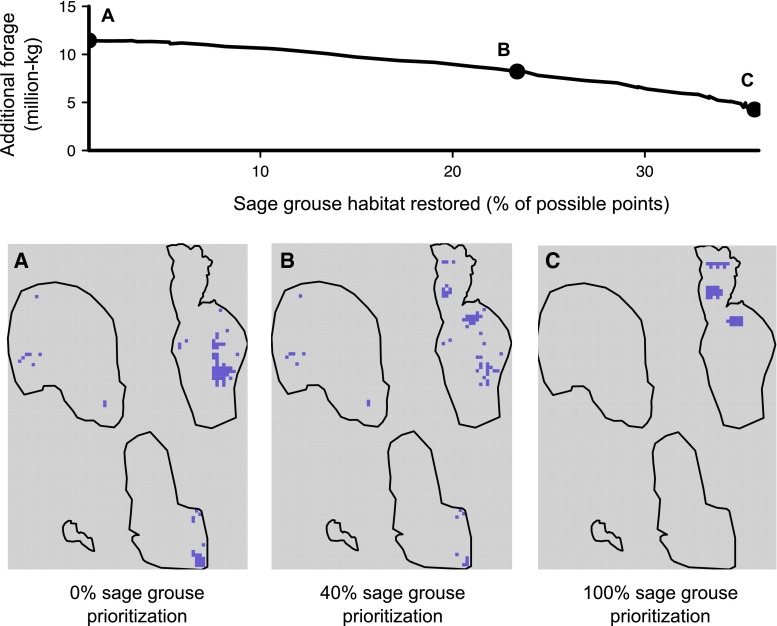
Baseline efficiency frontier showing spatial configuration of treated juniper at various weightings of sage-grouse habitat and forage production. Changing the prioritization of sage-grouse habitat restoration and forage production goals substantially affects the sites selected for juniper removal, shown in the three *inset graphs*. Complete sage-grouse habitat prioritization and complete forage production prioritization have entirely different selections of treatment sites

Juniper removal can benefit both sage-grouse habitat and cattle forage production, but outcomes depend on prioritization of goals. Juniper removal policies that prioritize forage production (i.e., selecting sites with the highest potential forage yield) result in little to no benefit for sage-grouse. Conversely, when juniper removal decisions are directed to improve sage-grouse habitat, substantial forage production benefits can accrue. In this case, tradeoffs between the two goals vary depending on the degree to which management objectives prioritize sage-grouse versus forage production. For instance, as the percentage of sage-grouse habitat restored increases, the relative gain in forage production declines (Fig. 5). These tradeoffs are linked directly to the spatial distribution of benefits on the landscape. Because each treatment site differs in terms of its potential benefit to sage-grouse and forage production goals, shifting prioritization of the two goals changes the network of sites selected for treatment on the landscape. In our study region, the two extremes of goal prioritization (100 % emphasis on sage-grouse goals versus 100 % emphasis on forage production goals) have entirely different selections of treatment sites (Fig. 5).

### Alternative Model Cases

**Fig. 6:**
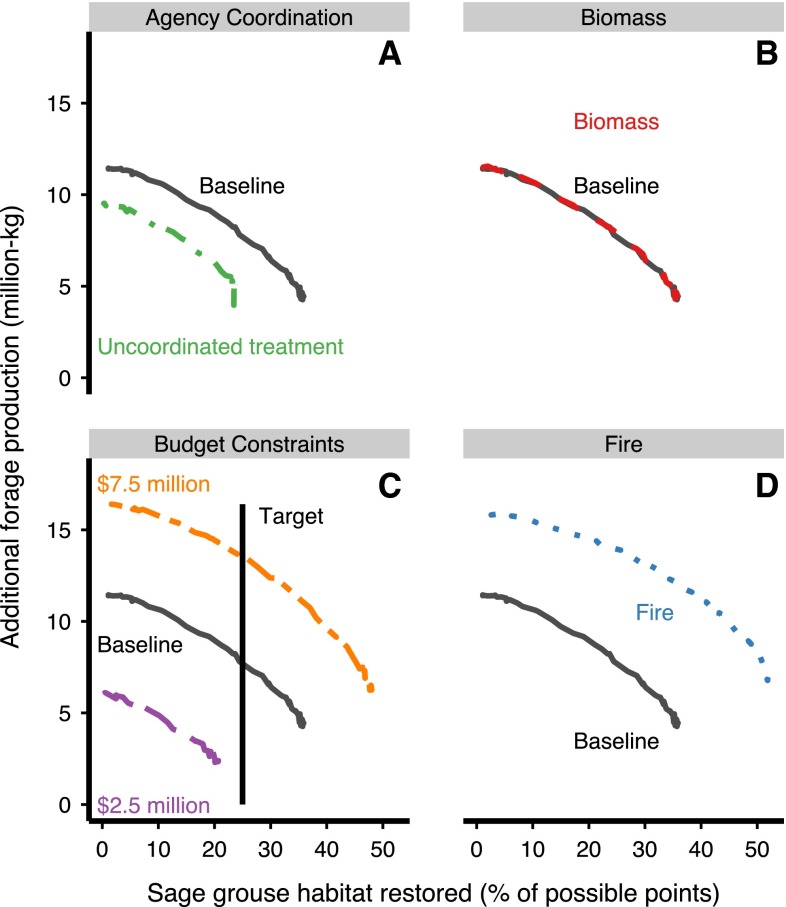
Tradeoff curves for alternative analysis cases of **a** varying the level of federal agency coordination in treatment; **b** including chipped juniper biomass as a resource to offset management costs; **c** adjusting budgetary constraints; and **d** including fire as a treatment method. In *each graph*, the baseline case is shown in *black*. An outward shift in the curve away from the origin indicates that more benefits can be achieved. In the “Budget Constraints” panel, the *vertical line* shows a target of 25 % sage-grouse habitat restoration, which is only achievable at two of the budgets shown

Our results suggest that agency coordination, budgetary constraints, and fire affect the amount of achievable benefits for ranching and sage-grouse conservation goals. However, the shape of the curves, which indicates the relationship between the two objectives, was constant (Fig. 6). We present the results for each case below.

#### Alternative Case 1: Lack of Agency Coordination

The degree of coordination among managing agencies substantially affected achievable benefits. Notably, when efforts to remove juniper are uncoordinated, the efficiency frontier decreases substantially in comparison with the baseline model (Fig. 6a) and reduces the potential benefits of juniper removal for the two goals.

#### Alternative Case 2: Biomass as an Additional Resource

We found that in 321 sites (out of 2193) the sale of wood chips to a local biomass plant (Fig. 2) could subsidize the cost of treatment by amounts ranging from $427 to $108,953. Although the biomass market can reduce treatment costs for some potential sites, this cost reduction (mean = $35,891) is not large enough compared to the cost of treatment (mean = $139,945) to allow for the treatment of additional sites. For this reason, the efficiency frontier does not expand in comparison with the baseline (Fig. 6b).

#### Alternative Case 3: Variable Budgets

Potential benefits of juniper removal changed in proportion to the available budget (Fig. 6c). A larger budget expanded the frontier of possible benefits, allowing for more sage-grouse habitat conservation as well as greater forage production. In comparison, smaller budgets reduced the potential benefits for both goals.

#### Alternative Case 4: Fire as a Treatment Method

When we included prescribed burns as a possible treatment option, the efficiency frontier expanded substantially compared to the baseline model (Fig. 6d). Because fire is a less costly juniper removal method, a much larger area could be treated for a given budget, which in our model yielded significantly greater forage production and sage-grouse habitat restoration.

## Discussion

For land managers in Northeastern California, multi-objective management of western juniper requires thoughtful prioritization of goals. Our results indicate that sage-grouse conservation and forage production goals may be complementary in some scenarios, but not without tradeoffs. Critically, prioritizing juniper removal decisions to improve forage production only produces a small increase in sage-grouse benefits. This tradeoff is directly related to the spatial distribution of benefits across the study region. Potential sage-grouse benefits are highest on land parcels with relatively dense juniper cover that are located in close proximity to lek locations. These benefits decay rapidly with increasing distance from a lek. Thus, treating a land parcel that is geographically isolated from lek locations would confer some amount of forage production benefits but no sage-grouse benefits. Overall, the model results indicate that juniper removal projects selected solely for rangeland benefits will not always benefit sage-grouse.

Our model also suggests that institutional constraints substantially alter potential benefits of juniper removal. While prescribed burns may be the most cost-effective method of juniper removal in California and many parts of the western U.S., bureaucratic and legal restrictions that seek to ensure safety may limit the use of this treatment option (Brunson and Evans 2005). Even in the absence of institutional constraints, it is unclear whether the use of prescribed fire would benefit sage-grouse. At least two long-term studies have reported negative effects of fire on sage-grouse nesting habitat (Nelle et al. 2000) and male lek attendance (Connelly et al. 2000), suggesting that land managers should exercise caution when using prescribed burns as a habitat restoration strategy.

Coordination among federal agencies working to control juniper can also increase achievable benefits for sage-grouse habitat improvement and forage production. However, the budgetary allocations that flow through different federal and state agencies can reduce incentives to cooperate in landscape-level approaches to juniper removal. While the SGI is novel in its broad coalition of public and private partners, project funding is often still allocated separately.

At present, budgets for juniper removal through the SGI are relatively large (NRCS 2012a). Although our model results indicate that sage-grouse and forage benefits scale with budget, selected targets for sage-grouse or forage might not be achievable at all budgets. For instance, a total program budget of $2.5 million is insufficient to achieve a goal of 25 % of the total benefits for sage-grouse populations (Fig. 6c). This could have considerable implications if biologically relevant sage-grouse benefits do not accrue without a minimum level of spending. Below this budget amount, only sub-optimal sites with limited potential benefit for sage-grouse would be restored. Such outcomes could result either from implementation issues or from overall budgetary reductions.

At a regional scale, our analysis shows that large scale initiatives can effectively manage juniper for both sage-grouse and cattle ranching goals. However, because the balance of prioritization between the two goals determines the range of potential shared benefits and overall complementarity, management may not meet multiple goals unless it is explicitly designed to do so. To avoid funding projects that have little or no benefit for sage-grouse, careful oversight and post-treatment evaluation are necessary.

## Electronic supplementary material

Below is the link to the electronic supplementary material.

Supplementary material 1 (DOCX 408 kb)
