# Supplementary material for: Western Juniper Management: Assessing Strategies for Improving Greater Sage-grouse Habitat and Rangeland Productivity
Source: Environ Manage. 2015 May 10;56(3):675–83. doi: 10.1007/s00267-015-0521-1 (PMC4527980; doi:10.1007/s00267-015-0521-1)
Supplement: Supplementary file 1 — Supplementary material 1 (DOCX 408 kb) [file 267_2015_521_MOESM1_ESM.docx]

**Online Appendix: Supplementary Information on Methods**

We used regression, geospatial analysis, imagery data of juniper cover, and field data for sage-grouse activity to derive values for benefits and costs of removing western juniper in the Modoc Plateau. The sections below provide further detail on the methodologies used in each step.

**A1. Estimating Forage Production Benefits from Removing Juniper**

Using herbaceous vegetation and juniper canopy cover data from Coultrap et al. (2008), we developed a forage production response model to estimate the effect of juniper canopy cover on herbaceous production. We adopted the log-linear functional form developed by Johnson et al. (1999) and then estimated forage production using environmental and geophysical data specific to our study area. The functional form of the estimated forage response function is:

$ln{(FP)}_{i}=Constant+ B_{1}\times{treated}_{i}+B_{2}\times{juniperper}_{i}+B_{3}\times{bffp}_{i}+B_{4}\times{julianday}_{i}+B_{5}\times{slope}_{i}+B_{6}\times{td}_{i}+B_{7}\times{paw0150}_{i}$

where *FP* is forage production in kilograms/hectare, *Constant* is the intercept, *treated* is a binary variable for whether a cell (*i*) experienced juniper removal, *juniperper* is the percent juniper canopy cover prior to treatment, *bffp* is the Julian date on which frost-free period begins, *julianday* is the Julian day of sampling, *slope* is the representative slope of the sampled plot, *td* is the temperature difference between the mean warmest month temperature and mean coldest month temperature (°C), and *paw0150* is the measured plant available water in the upper 150-mm of soil. We explored additional variables (e.g., aspect, elevation, minimum temperature, and maximum temperature) as potential predictors of forage production, but we chose to omit them from the model because they explained little additional variance in forage production and/or were highly correlated with the predictors we did include. All sites are indexed according to *i*.

|  | (1) | (2) |
| --- | --- | --- |
| Variable | Coeff | SE |
|  |  |  |
| treated | 0.625** | (0.160) |
| juniperper | -0.0161** | (0.00443) |
| bffp | -0.0406** | (0.0133) |
| julianday | -0.00503 | (0.00384) |
| slope | 0.0441 | (0.0244) |
| td | -0.233** | (0.0640) |
| paw0150 | 0.0403* | (0.0197) |
| Constant | 15.38** | (2.467) |
|  |  |  |
| Observations | 97 |  |
| Adjusted R-squared | 0.402 |  |
| F test | 10.20 |  |

Standard errors in parentheses

** p<0.01, * p<0.05

**Table A.1** Forage production regression model results of estimating equation (1) by ordinary least squares. *Treated*, *juniperper*, *bffp*, *td*, and the constant were significant at the 1% level while *paw0150* was significant at the 5% level. The equation had an adjusted R^2^ of 0.402 and F-statistic significant at the 1% level

**A2. Determining Benefits for Sage-Grouse Habitat**

To estimate sage-grouse benefits following juniper removal, we developed a dispersal index that predicted the relative likelihood of sage-grouse colonizing and occupying the cell given conversion from juniper to sagebrush. The index was largely based on lek location data obtained from the California Department of Fish and Wildlife and the Oregon Department of Fish and Wildlife. The rationale for using lek data was two-fold. First, established protocols exist for counting male sage-grouse at lek sites (Connelly et al. 2004). Additionally, leks and the areas surrounding leks are ideal for these analyses because they encompass critical breeding grounds (Autenrich 1985; Connelly et al. 2004) and are generally centered among seasonal use areas (Coates et al. 2013). Nearly all leks were counted four times per season with a maximum count recorded. Although lek counts are a widely used technique for estimating sage-grouse population sizes, we acknowledge that some limitations may exist, including observer bias and spatiotemporal variation in lek attendance (Walsh et al 2004).

Three steps were used to calculate the dispersal index. First, we accounted for the relative density of sage-grouse across the landscape by calculating a breeding density index using lek coordinates and count data (number of males attending leks). Only leks active within the last 10 years and within the boundaries of our study area were used for this analysis. We used kernel density estimation (Silverman 1986) on the lek locations, weighting each lek by 5-year average peak counts. The smoothing parameter was estimated using likelihood based cross-validation (Horne and Garton 2006) within Geospatial Modeling Environment (Beyer 2012) and Program R (‘ks’ package, Duong 2012).

Second, we accounted for seasonal space use patterns based on distance to nearest lek sites, which we refer to as a distance index. Because the probability of occurrence at seasonal areas is not likely to be a linear relationship with the Euclidean distance from treatment area to lek, we adopted the values used to generate a non-linear space use response curve previously published in Coates et al. 2013 (Fig. A1). Although those values were derived from an analysis conducted within Mono County (approximately 400 km south of our study site), the curve represents variation from multiple subpopulations and can serve as a baseline in areas where telemetry data are lacking (Coates et al. 2013). The curve was generated from averaged seasonal utilization distributions (30-m grid cell) based on nearly 12,000 sage-grouse locations from radio telemetry methods, as described in Coates et al. 2013. After obtaining space use values, we calculated the inverse of the curve to create the distance index based on grid cell proximity to the nearest lek site. Grid cells at lek sites were rescaled to a value of one. As the distance from lek increased, the relative value of each grid cell decreased rapidly until approximately 5 km, at which the curve flattens (Fig. A1). Lastly, for each grid cell we averaged the two indices (density and distance) and rescaled between 0 and 1 to reflect the dispersal index using spatial analysts tools in ArcGIS 10.1 (Environmental Systems Research Institute, Redlands, CA).

**A3. Nested Geospatial Analysis**

We divided our study region into a grid of 4 km^2^ cells, which we consider the “decision units.” This size is comparable to the median area of recent juniper treatment projects on the Modoc Plateau (Sage Steppe Ecosystem Restoration Strategy Database; http://ltdl.wr.usgs.gov/SSERS/). We modeled treatment decisions (i.e. treat or not) for these grid cells. However, to assess site attributes and estimate the costs and benefits of removing juniper, we further divided each 4 km^2^ cell into 400 square cells of 1 ha (100 m^2^). We assessed geospatial attributes (i.e., juniper cover, forage production potential, sage grouse habitat suitability) at this finer scale to model treatment outcomes. However, because treatment decisions are made at the 4 km^2^ scale, we aggregated 100 m^2^ cells to the 4 km^2^ scale by simply adding the treatment cost and benefit of all 100 m^2^ cells within each 4 km^2^ cell. We identified cells that were partially or completely covered by water at the 100 m^2^ scale. For those cells partially covered by water, we scaled treatment costs and potential benefits proportionately to the percentage of dry land. We assigned costs and benefits of zero to cells that were completely within water bodies.

**A4. Alternative cases**

***Alternative case 1: Biomass as an additional resource***. We considered a scenario in which treatment costs of removing juniper can be offset by selling chipped juniper biomass to the Greenleaf Power Plant (Wendel, CA) (Fig. 2), the only power plant close enough to our study region to purchase juniper chips. Our interviews with juniper treatment practitioners and a Greenleaf Power Plant manager revealed that the cost of transporting juniper biomass is a primary limiting factor. Based on these interviews as well as a review of the literature, we altered the calculation of weighted cost-effectiveness for each cell (*i*) to include revenues from selling juniper biomass ($B_{i_{biomass}}$) as a function of distance to the biomass plant ($d_{i}$) and juniper canopy cover $(c_{i})$:

| $Z_{i}= \frac{\left[ \left( W_{forage}* B_{i_{forage}} \right)+ \left( W_{habitat}* B_{i_{habitat}} \right)*f \right]}{C_{i}-B_{i_{biomass}}}$ | (5) |
| --- | --- |
| ${where B}_{i_{biomass}}= \left\{ \begin{aligned} 48*\frac{c_{i}}{2}*\left( 1-\left( \frac{d_{i}}{120} \right) \right) \\ \end{aligned} \begin{matrix} where d_{i}<60 miles \\ where d_{i}>60 miles; \$0 \end{matrix} \right.$ | (6) |

We calculated the travel distance between treatment sites and the power plant using the ArcGIS Network Analyst extension (ESRI 2012) and the “U.S. and Canada Detailed Streets” dataset (ESRI 2010), which provides extensive coverage of our study region, including local dirt roads. Based on interviews, we used $48 as the price per ton of delivered juniper chips and 120 miles as the break-even distance for transportation. We estimated the number of tons of juniper chips produced by treating a given site by multiplying the percent juniper canopy cover of the cell by the constant 0.5 (BLM 2010).

***Alternative case 2: Fire as a treatment method***. We also considered an alternative scenario in which prescribed fire is a treatment option at the cost and site characteristics provided in Table A2. Despite considerable interest in fire as a low-cost tool, it is not used extensively due to liability issues. Because prescribed fire is viable at <20% juniper cover, in this model it replaced all hand treatment and some mechanical treatment.

***Alternative case 3: Variable budgets***. We performed a sensitivity analysis on the budget value from the baseline case ($5 million) by running the model assuming total budgets of $2.5 million and $7.5 million.

***Alternative case 4: Lack of agency coordination****.* The baseline model assumed that all major land management groups collaborate perfectly by sharing financial resources and carrying out projects across jurisdictional boundaries. In reality, funding is often restricted to specific ownership groups. For example, private landowners typically pay for treatment on their own land, while the U.S. Forest Service (USFS) obtains funding specifically for Forest Service land. Moreover, USFS and Bureau of Land Management (BLM) land may not be adjacent to private lands that are treated. We altered the baseline case to reflect the absence of collaboration between major land management groups in the region, which includes BLM, USFS, and private landowners. The budget was divided in proportion to the land area each group manages (Fig. A2) and all juniper treatment decisions were made independently. The model was run separately and the benefits summed across the three groups.

|  | **Baseline Case** | | **Fire Case** | |
| --- | --- | --- | --- | --- |
| **Treatment Method** | **Hand** | **Mechanical** | **Fire** | **Mechanical** |
| *Juniper canopy cover* | 0-10% | 10-30% | 0-20% | 20-30% |
| *Slope* | <30% | <30% | <30% | <30% |
| *Cost/ha* | $100 | $300 | $75 | $300 |

**Table A.2** Assignment of treatment costs based on site attributes. Data were collected through semi-structured interviews with stakeholders, including representatives from private consulting firms, federal land management agencies, and cooperative extension

| Analysis | Description | | Benefits | Costs | Constraints |
| --- | --- | --- | --- | --- | --- |
| *Baseline*  *Case* | Assess optimal selection of juniper removal sites to maximize benefits for sage-grouse habitat and forage production, within a budget constraint. | | 1. Increased forage production  2. Improved sage-grouse habitat | Juniper removal cost, based on canopy cover and treatment type:  a) Hand treatment: $100/ha (interviews indicated $11-$250/ha)  b) Mechanical treatment: $300/ha (interviews indicated $180-$400/ha) | 1. Hand treatment only available for sites with 10-15% canopy cover  2. Mechanical treatment available for sites with 10-30% canopy cover  3. Maximum canopy cover of 30% for any treatment method  4. $5 million budget |
| *Alternative Cases* | | | ***Changes from Base Case*** | | |
| *Case 1:*  *Agency*  *Coordination*  (Fig. 6a) | | Include variable coordination levels among land managers (BLM, USFS, private landowners) | *Same as Baseline Case* | *Same as Baseline*  *Case* | Budget divided into portions so that agencies only treat juniper on land under their ownership rather than pooling funds to treat the optimal sites |
| *Case 2:*  *Biomass as a Resource*  (Fig. 6b)  *Case 3:*  *Budget Constraints*  (Fig. 6c)  *Case 4:*  *Fire as Treatment Option*  (Fig. 6d) | | Treatment costs offset by selling chipped juniper biomass to a local power plant for power production (Fig. 2)  Analysis using varying budgets of total funds available for treatment  Include fire as a juniper treatment option | Additional benefits accrue from sale of juniper biomass ($48/ton for sites <60 miles of a biomass plant)  *Same as Baseline Case*  *Same as Baseline*  *Case* | *Same as Baseline*  *Case*  *Same as Baseline*  *Case*  Additional treatment option of controlled burning at $75/ha (interviews indicated $50-$100/ha) | Biomass benefits only accrue for sites within travel radius of existing biomass plant  Variable budget availability, including:  a) $2.5 million  b) $5 million  c) $7.5 million  1. Fire treatment available for sites with 0-20% canopy cover  2. Mechanical treatment available for sites with 20-30% canopy cover |

**Table A.3** Summary of model formulation and analysis, including baseline case and alternative cases. Benefits, costs, and constraints are detailed for each case. For the alternative cases, changes in analysis or formulation from the baseline case are noted


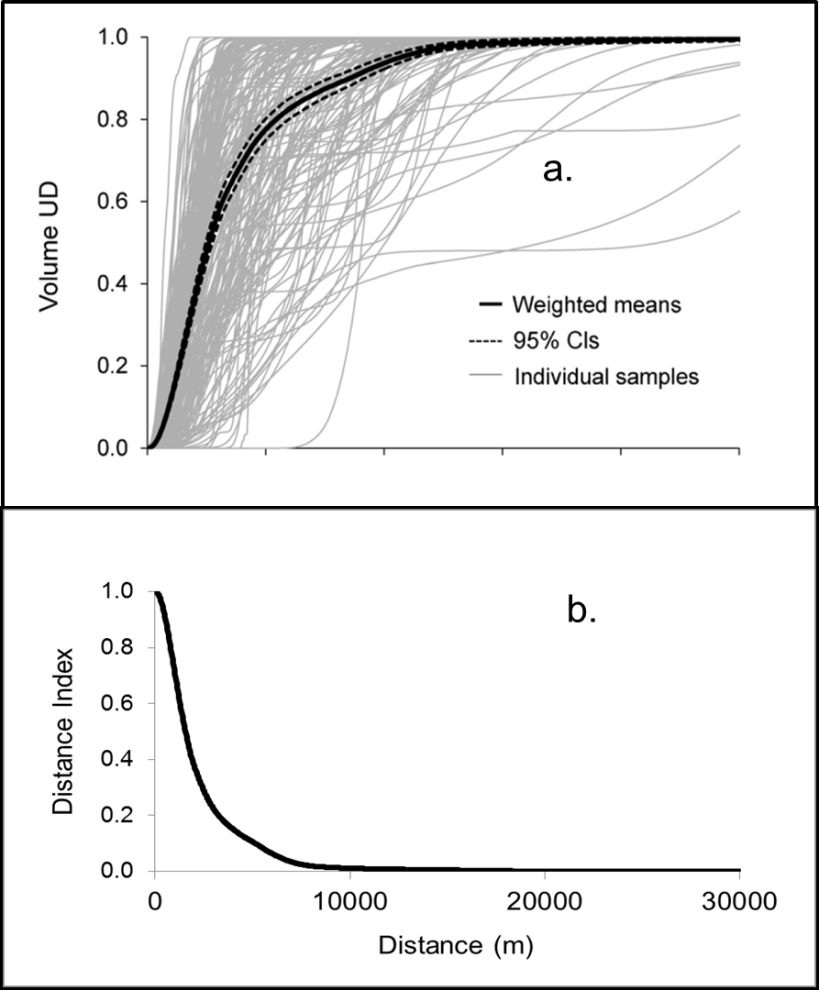


**Figure A.1** Response curves for (a) volume of utilization distribution and (b) distance index as the inverse of vUD to represent the relative probability of occurrence. Reproduced with permission from Coates et al. 2013


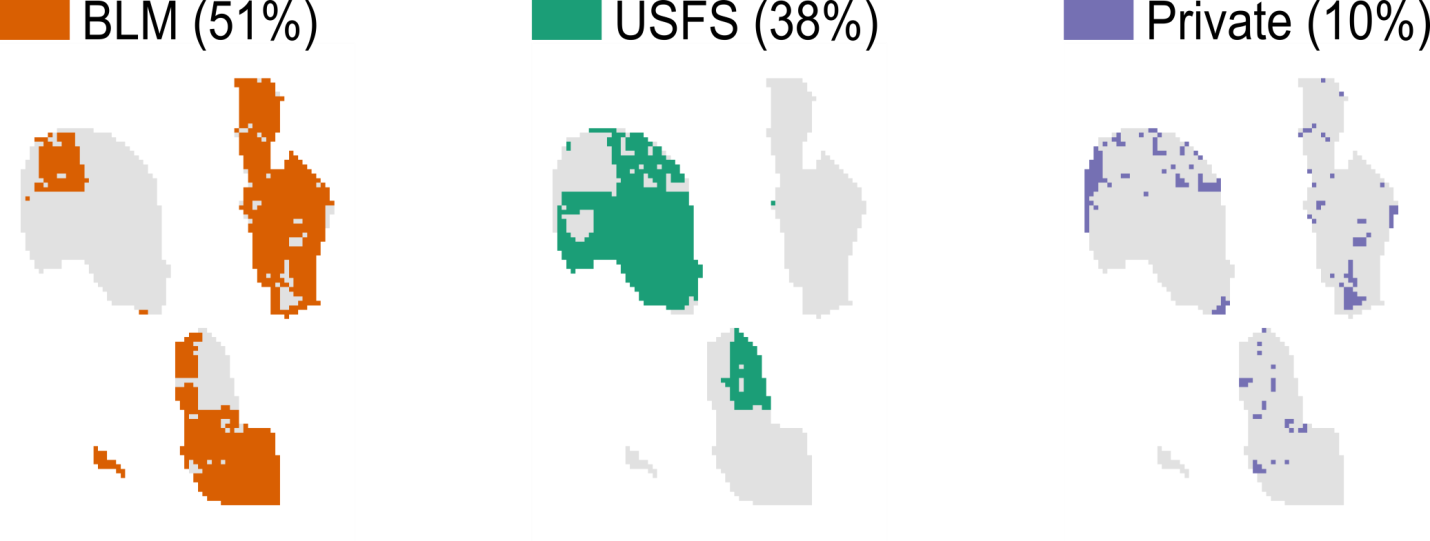


**Figure A.2** Distribution of potential juniper treatment sites by category of management group: Bureau of Land Management (BLM), United States Forest Service (USFS), and private landowners

**Additional references**

Autenrieth R (1985) Sage grouse life history and habitat management. Rangeland Fire Effects: A Symposium. U.S. Bureau of Land Management, University of Idaho, Boise, Idaho, p 52

Beyer HL. 2012. Geospatial Modelling Environment (Version 0.7.2.0). http://www.spatialecology.com/gme.

BLM (2010) *Bureau of Land Management, Northeast California Resource Advisory Council: Field Tour and Meeting*. U.S. Bureau of Land Management, Alturas, CA

Coates PS, Casazza ML, Blomberg EJ, Gardner SC, Espinosa SP, Yee JL, Wiechman L, Halstead BJ. 2013. Evaluating greater sage-grouse seasonal space use relative to leks: Implications for surface use designations in sagebrush ecosystems. The Journal of Wildlife Management 77: 1598-1609.

Connelly, J. W., S. T. Knick, M. A. Schroeder, and S. J. Stiver. 2004. Conservation assessment of greater sage-grouse and sagebrush habitats. Western Association of Fish and Wildlife Agencies, Cheyenne, Wyoming, USA.

Coultrap DE, Fulgham KO, Lancaster DL, Gustafson J, Lile DF, George MR (2008) Relationships between western juniper (*Juniperus occidentalis*) and understory vegetation. Invasive Plant Sci Manag 1:3–11.

Duong T. 2012. ks: Kernel smoothing. R package version 1.8.10. http://CRAN.R-project.org/package=ks.

Environmental Systems Research Institute (ESRI) (2010) U.S. and Canada Detailed Streets, Compiled by Tele Atlas North America (2005), Inc. Redlands, CA

Environmental Systems Research Institute (ESRI) (2012) *ArcGIS 10.1*. Environmental Systems Research Institute, Redlands, CA

Horne JS, Garton EO. 2006. Likelihood cross-validation versus least squares cross-validation for choosing the smoothing parameter in kernel home-range analysis. Journal of Wildlife Management 70: 641-648.

Johnson P, Gerbolini A, Ethridge D, et al (1999) Economics of redberry juniper control in the Texas rolling plains. J Range Manag 569–574.

R Core Team (2014) R: A Language and Environmental for Statistical Computing. R Foundation for Statistical Computing, Vienna, Austria.

Silverman BW. 1986. Density estimation for statistics and data analysis. Chapman & Hall, London, United Kingdom.

Walsh DP, White GC, Remington TE, Bowden DC (2004) Evaluation of the lek-count index for greater sage-grouse. Wildlife Society Bulletin 32:56–68.
